# Chromosome-level genome assembly of *Platycarya strobilacea*

**DOI:** 10.1038/s41597-024-03107-4

**Published:** 2024-03-05

**Authors:** Huijuan Zhou, Xuedong Zhang, Hengzhao Liu, jiayu Ma, Fan Hao, Hang Ye, Yaling Wang, Shuoxin Zhang, Ming Yue, Peng Zhao

**Affiliations:** 1 Xi’an Botanical Garden of Shaanxi Province, Institute of Botany of Shaanxi Province, Shaanxi Academy of Science, Xi’an, Shaanxi 710061 China; 2 Key Laboratory of Resource Biology and Biotechnology in Western China, Ministry of Education, College of Life Sciences, Northwest University, Xi’an, Shaanxi 710069 China; 3 College of Forestry, Northwest A&F University, Yangling, Shaanxi 712100 China

**Keywords:** Plant evolution, Ecological genetics

## Abstract

*Platycarya strobilacea* belongs to the walnut family (Juglandaceae), is commonly known as species endemic to East Asia, and is an ecologically important, wind pollinated, woody deciduous tree. To facilitate this ancient tree for the ecological value and conservation of this ancient tree, we report a new high-quality genome assembly of *P. strobilacea*. The genome size was 677.30 Mb, with a scaffold N50 size of 45,791,698 bp, and 98.43% of the assembly was anchored to 15 chromosomes. We annotated 32,246 protein-coding genes in the genome, of which 96.30% were functionally annotated in six databases. This new high-quality assembly of *P. strobilacea* provide valuable resource for the phylogenetic and evolutionary analysis of the walnut family and angiosperm.

## Background & Summary

*Platycarya strobilacea* belongs to the walnut family (Juglandaceae), is commonly known as a species endemic to East Asia, and is an ecologically important, wind pollinated, woody deciduous tree^1–3^. It is known as a tertiary relict tree, and is widely native to East Asian (China, Japan, Korea, and Vietnam) in the sunny mountainous regions^1–5^. *P. strobilacea* is considered to have the widest geographic distribution in the genus *Platycarya*, mainly occurring in East Asia^3,6,7^. It is also known for its systematic and evolutionary ancient morphology, such as its unique systematic position in Juglandaceae^2,4^ wingnuts and its bisexual inflorescence aggregated on the apices of branches^5–8^. Based on morphological and molecular evidence, *P. strobilacea* is considered to occupy a unique phylogenetic position in a sister group between Engelhardioideae and Juglandoideae^5,9,10^. Species within the Juglandaceae can be divided into three sub-families, namely Juglandoideae, Engelhardioideae, and Rhoipteleoideae, as supported by previous studies^6,11^. The fossil data, morphology, and molecular data have conflicting results regarding *P. strobilacea*’s phylogeny in Juglandaceae^6,9–12^. *P. strobilacea* is considered a sister group between *Carya* and *Cyclocarya* and the most of ancient wingnut groups are closely related to *Cyclocarya* within the subfamily Juglandoideae^6,11–13^.

*P. strobilacea* is an ancient tree, and it has the widest distribution in the genus *Platycarya* in Eastern Asia, especially in subtropical China^14^. It previously occupied large range across the Northern Hemisphere according to the fossil record, but now only survives only in East Asia^7,14,15^. The bark, root bark, leaves, and fruit infructescence of *P. strobilacea* contain raw materials used for extracting tannin extraction. The bark can also be utilized for its fibers, the leaves can be used as pesticides, the roots and old trees contain aromatic oil, and the seeds contain oil which can be extracted. The morphology, biogeography, and population genetic of *P. strobilacea* have been described^3,5,12^. Previous studies on *Platycarya* detected a significant population structure and the multiple glacial refugia across most of the current geographic distribution range in China using chloroplast DNA and nuclear SNPs data^2,14^. The complex evolutionary history of *P. strobilacea* indicates that its morphology and genome might be influenced by climate change and environmental adaption. To meet demand for improved ecological conservation biology of this important tree, the high-quality whole genome sequence data is an essential genetic resource for this ecologically woody deciduous tree^2,4,9,14,15^. Useful genetic and genomic data of species in the Juglandaceae subgroup were recently published^4,16–21^.

Here, we report a new high-quality chromosome-level genome assembly of *P. strobilacea* (NWU2021168). The whole genome of *P. strobilacea* was generated using short and long read sequencing data generated using the Illumina Hiseq, PacBio single-molecule real-time sequencing technology, and Hi-C platforms. We produced transcriptome expression profiles of different tissues related to flowering and stress genes in *P. strobilacea*. The genome sequence of *P. strobilacea* reported here is a new genomic resource for the genetic study of *P. strobilacea*, for genome evolution analysis in the walnut family and Angiosperms, and for exploring its potential ecological values.

## Methods

### Sample and whole genome sequencing

**Fig. 1:**
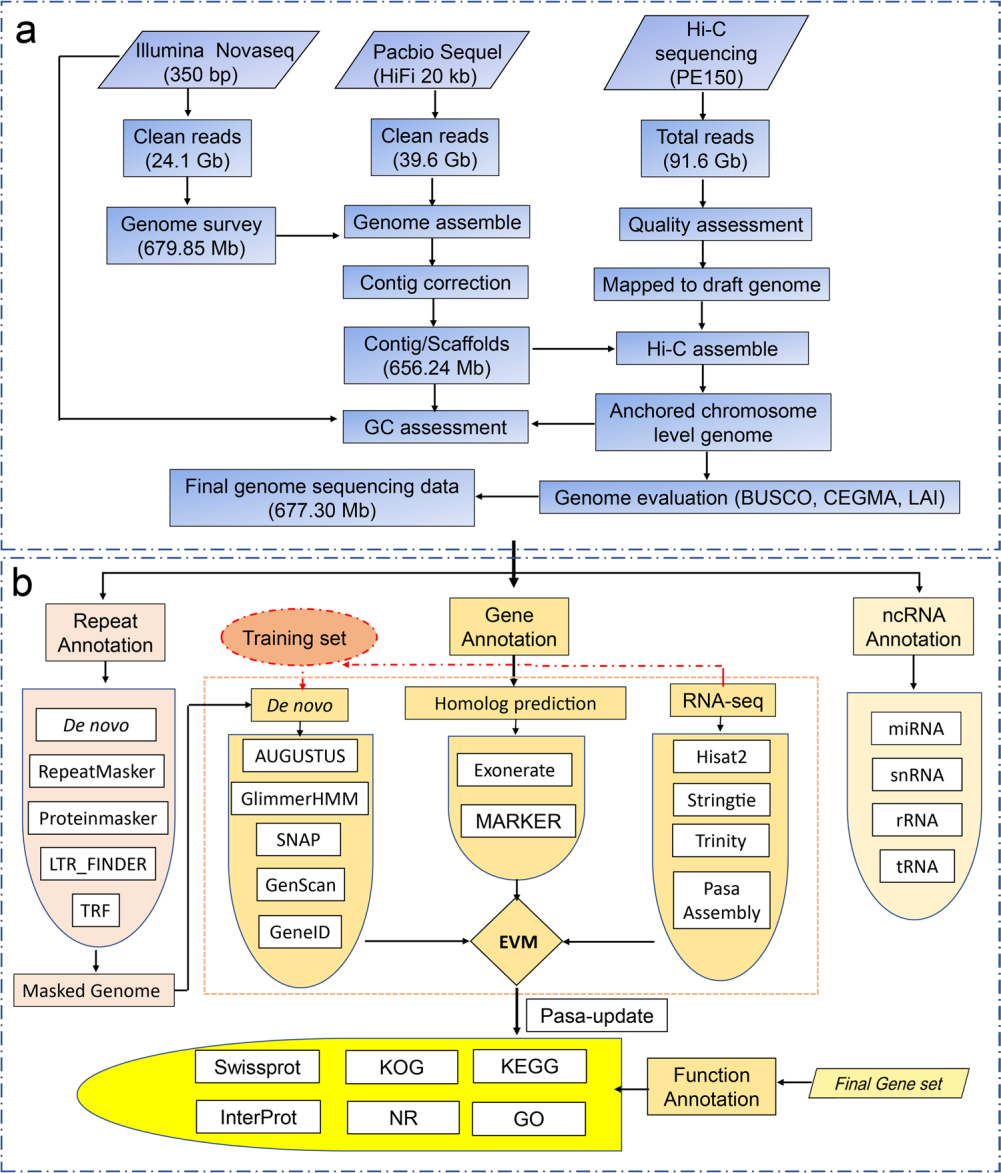
The *Platycarya strobilacea* genome sequencing assembly and annotation pipeline. (**a**) Genome assembly with a combination of Illumina, Pacbio, and Hi-C sequencing technologies. (**b**) The *Platycarya strobilacea* genome annotation workflow, including repeat annotation, gene annotation, and noncoding RNA (ncRNA) annotation.

In 2021, we collected young and heathy leaves from a single individual of *P. strobilacea* (genotype NWU2021168), growing in Qinling Mountain, Shaanxi, China (altitude: 1268 m, 33°68′N, 107°35′E). Total high-quality genomic DNA of NWU2021168 was prepared from the fresh leave samples using a kit (TIANGEN, Beijing, China). A DNA library (350 bp) was constructed based on short-read data obtained from the Illumina Novaseq 6000 platform (Illumina, San Diego, CA, USA) for the genome survey. PacBio Sequel II HiFi long-read (20 kb) libraries were constructed and then sequenced for long reads (Novogene, Beijing). The Hi-C library was prepared and then sequenced based on the Illumina Novaseq 6000 platform (Illumina, San Diego, CA, USA) for the chromosome-level genome sequencing. The genome sequencing was completed using a combination of Illumina, Pacbio, and Hi-C sequencing technologies (Fig. 1a). After filtering out the low-quality reads, we obtained a total of 155.3 Gb (240.3 ~×) of clean reads, including 24.1 Gb (35.4×) of Illumina reads, 39.6 Gb (59.6×) of PacBio long-reads, and ~91.6 Gb (145.4×) of the Hi-C reads (Table 1).

**Table 1:** Summary of sequencing data of *Platycarya strobilacea*.

Platform	Insert Size (bp)	Raw reads	Coverage(X)	N50
Illumina Hiseq	350	677,303,888	35.4	1328
Pacbio Sequel	20,000	13,204,805	59.6	95,562
Hi-C	350	45,791,698	145.4	45,791,698
Total	39.15	39.15	240.3	/

### Genome *de novo* assembly and assessment

**Fig. 2:**
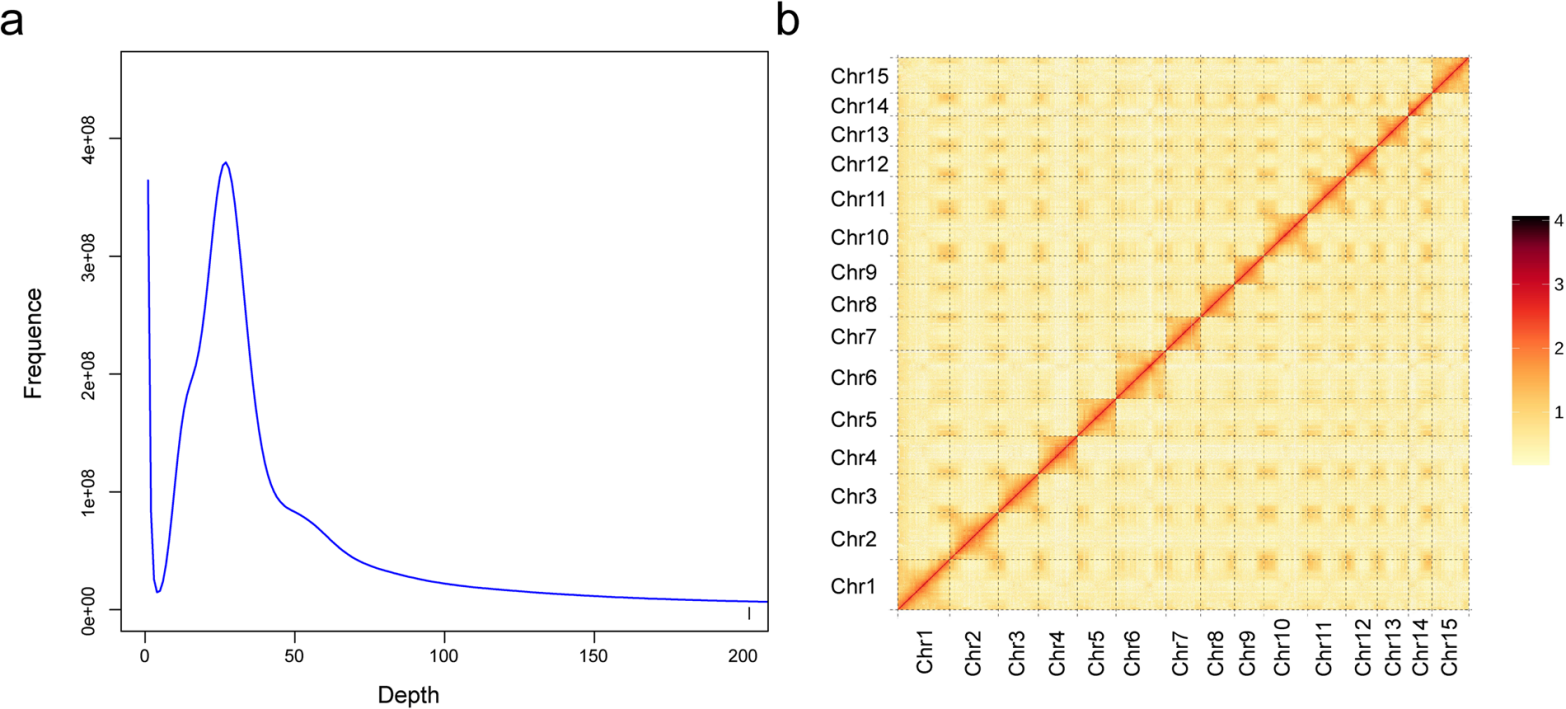
Genome size estimation and Hi-C heatmap of *Platycarya strobilacea*. (**a**) Genome size estimation by 17-K-mer analysis of *P. strobilacea*. (**b**) Heatmap of chromosomes of *Platycarya strobilacea* after Hi-C assisted assembly.

**Fig. 3:**
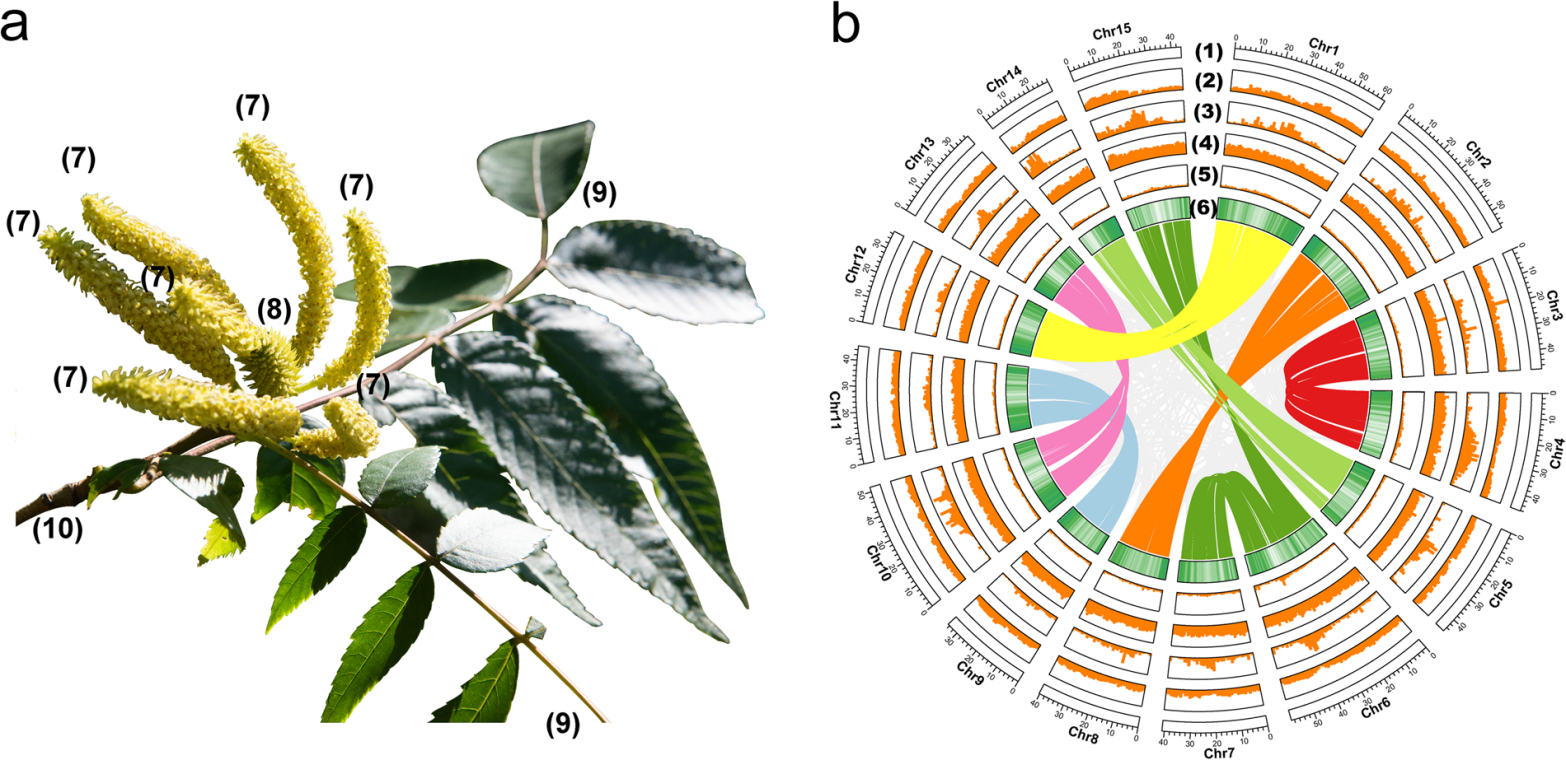
Morphology and genome map of *Platycarya strobilacea*. (**a**) morphology of *P. strobilacea* female flowers (7), male flowers; (8), female flowers (catkins); (9), leaf; (10), branch. The black bar represents 1.5 cm. (**b**) Circos map of the *Platycarya strobilacea* genome assembly. (1), gene density; (2) GC ratio; (3) TE density; (4) *Gypsy*; (5) *Copia*; (6) syntenic relationships among chromosomes.

The assembly of the whole genome of *P. strobilacea* and the subsequent assessment followed the pipeline (Fig. 1a). The raw reads of Illumina were evaluated with SOAPnuke v1.5.6^22^. We generated the 17-*K*-mer statistics of the sequencing reads from short libraries (350 bp) using k-mer methods. The genome size was estimated using means of 17-*K*-mer statistics (Fig. 2a)^23^. The estimated genome size of was about 677.30 Mb, and the proportion of GC content and the genome heterozygosity rate were determined to be approximately 34.12% and 1.13%, respectively (Table 1). *De novo* assembly of *P. strobilacea* was performed using the software Falcon v1.87^24^. Then, the sequencing reads from PacBio and Hi-C were mapped to our genome assembled scaffolds using the program BWA-aln^25^. Based on the Hi-C sequencing reads, the scaffolds were anchored to 15 pseudomolecules using LACHESIS^26^. The interaction heatmap of *P. strobilacea* chromosome pairs was produced using the software HiC-pro (Fig. 2b)^27^. Using the Hi-C mapping technology, the scaffolds were further anchored onto fifteen chromosomes that covered ~98.43% of the assembled sequences (Fig. 3). The final genome assembly was 677.30 Mb with an N50 of 43.67 Mb (Tables 1 and 2). Self-alignment analysis found that the duplications were present within a chromosome (Fig. 3b). The lengths of the fifteen assembled chromosomes of *P. strobilacea* ranged from 19,447,442 bp to 61,544,683 bp, with an average length of 42,331,493 bp (Fig. 3b).

**Table 2:** Statistical summary of the *Platycarya strobilacea* genome assembly and annotation.

Genomic feature	Value
Total genome size (bp)	677,303,888
N50 contig length (bp)	13,204,805
N50 scaffold length (bp)	45,791,698
GC Content (%)	39.15
Size of LTR (bp)	257,450,664
Size of DNA transposons (bp)	7,917,961
Size of total repeat sequences (bp)	297,372,221
Protein-coding gene number (*n*)	32,246
Mean coding sequence length (bp)	1,175
Mean exons per gene (*n*)	4.99
Mean exon length (bp)	235.46
Mean intron length (bp)	902.18
BUSCO completeness (%)	98.43

**Fig. 4:**
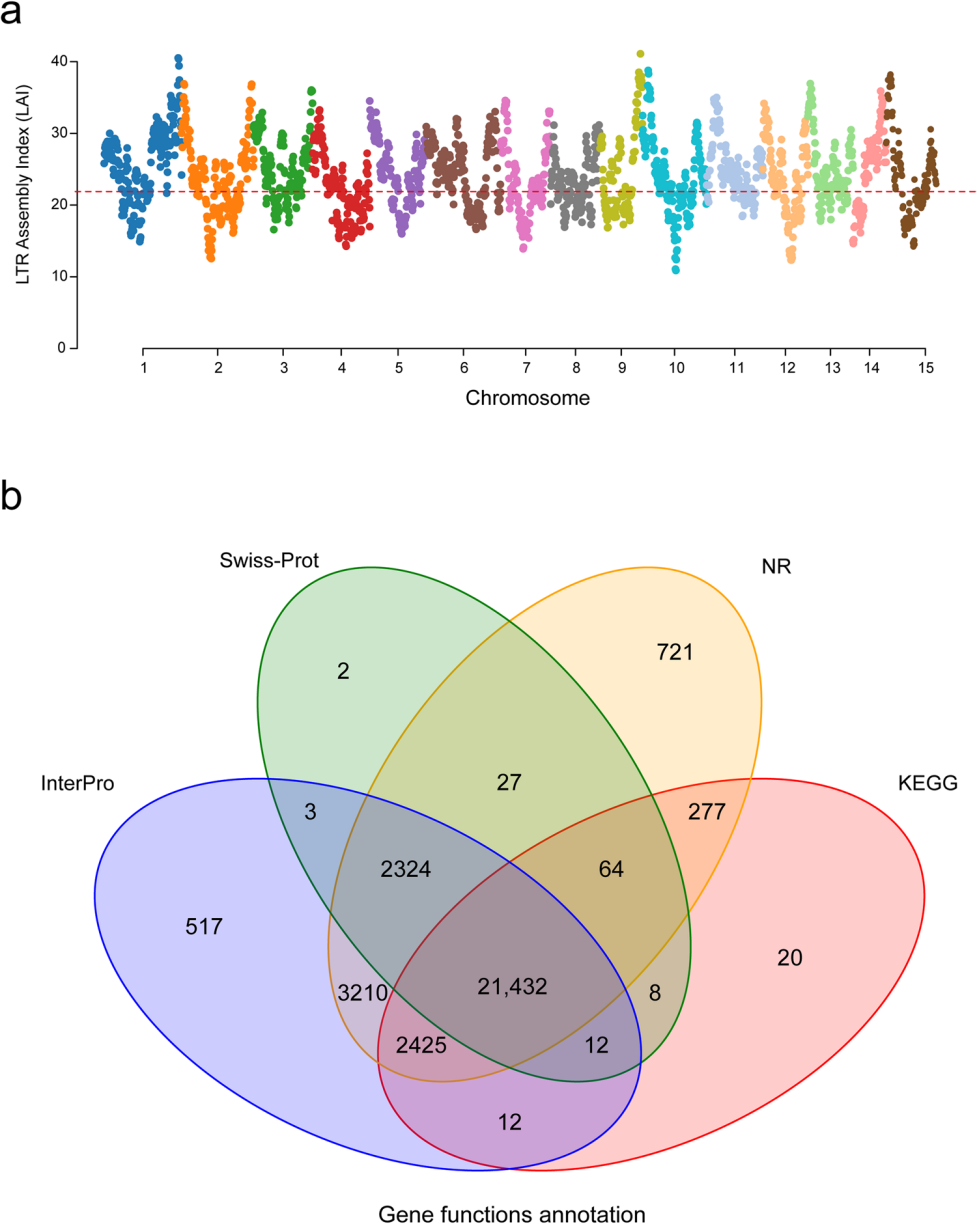
Assembly Index LAI assessment and gene function annotations of assembled *Platycarya strobilacea* genome. (**a**) Assembly Index LAI assessment for each assembled *P. strobilacea* chromosome. The average LAI is about 21.97, indicating the high quality of our assembly. Dashed line (LAI = 21.97) indicates the gold standard quality level of the assembly. (**b**) Venn diagram showing the shared and unique genes between the four gene functions annotation databases. Swiss-Prot = Swiss Institute of Bioinformatics and Protein Information Resource, InterPro = Protein sequence analysis and classification, NR = non-redundant, and KEGG = Kyoto Encyclopedia of Genes and Genomes.

The final completeness of the *P. strobilacea* genome assembly was evaluated using BUSCO v3.0.2 software^28^. We identified a total of 1,614 BUSCO groups, 1,598 (99.0%) complete BUSCOs, 8 fragmented BUSCOs, 129 duplicated BUSCOs, and 1,469 single copy BUSCOs in the NWU2021168 *P. strobilacea* assembly. Based on the CEGMA (Core Eukaryotic Genes Mapping Approach), 248 core eukaryotic genes (93.95%) were verified in the NWU2021168 assembly. We aligned the Illumina short read data (24.0 Gb) with our completed genome assembly, and 98.53% of the clean reads were mapped. The LAI (assembly index) of our *P. strobilacea* genome was 21.97 (Fig. 4a). These assessments validated the quality of the NWU2021168 assembly, showing that the *P. strobilacea* genome assembly is of good quality in both genic and intergenic regions.

### Genome annotation of protein-coding genes and repeats

Genome annotation was predicted using multiple methods, including transcriptomic data, de novo prediction, and homology-based annotation methods^19^. The details of genome annotation follow the pipeline are shown in Fig. 1b. To ensure accurate gene annotation, RNA sequences from eight tissues (female flower, male flower, mix female and flower inflorescence, axillary bud, new branch, stem, stem bark, and leaf) were used to annotate genes using the software AUGUSTUS (Table 3)^29^. These eight tissues were collected from the individual of *P. strobilacea* (genotype NWU2021168), which was subjected to whole-genome sequencing (some tissues showed in Fig. 3a). For transcriptome sequencing, we extracted RNAs from three biological duplications from each tissue, and then each of the three RNAs were mixed into one for RNA sequencing using Illumina Hiseq 2500 platform (Illumina, San Diego, CA, USA). We obtained a total of 369,124,704 clean data from eight tissues. The average amount of clean sequencing data was 46,140,588 bp with clean data ranging from 44,367,760 bp (stem bark) to 47,703,130 bp (mix female and flower inflorescence). A mean mapped clean read rate was 90.99% with the mapped rate ranging from 69.78% (stem bark) to 95.27% (stem), respectively (Table 3). The gene structure was annotated for protein-coding genes with reference to four species (*Juglans regia*, *Juglans sigillata*, *Carya illinoinensis*, and *Castanea mollissima*) using Exonerate v2.2.0^30^ for homology-based annotation. The final genome annotation of the protein-coding genes was determined using the software MAKER2^31^. We estimated the final protein-coding genes for functional annotation using six databases, including SwissProt^32^, Nr^33^, KEGG^34^, InterPro^35^, GO^36^, and Pfam^37^ databases, respectively (Fig. 4b and Table 4). Combining the multiple methods, we detected a total of 32,246 protein-coding gene models from the *P. strobilacea* NWU2021168 genome, with a mean coding sequence (CDS) length of 1,175 bp, an average exon length of 235 bp, and a mean of five exons per gene (Table 1). Among the 32,246 predicted genes, there were 30,480 (94.52%) genes annotated in the Nr database, 29,935 (92.83%) genes were annotated in InterPro, 24,250 (75.20%) genes were annotated in KEGG, 23,644 (73.32%) genes were annotated in Pfam, and 18,140 genes were annotated in GO database (Table 4), respectively.

**Table 3:** Statistical summary of transcriptome sequencing data from eight tissues for the *Platycarya strobilacea* genome annotation.

Order	Tissues	Clean reads (bp)	Reads mapped (%)
1	Axillary bud	45,885,334	94.32%
2	Leaf	45,023,197	94.99%
3	New branch	47,104,404	94.44%
4	Stem bark	44,367,760	69.78%
5	Stem	44,946,959	95.27
6	Female flower	47,635,226	90.16%
7	Male flower	46,458,694	95.12%
8	Mix female and male flower	47,703,130	93.82%

**Table 4:** Statistical summary of the annotation of the *Platycarya strobilacea* genome using six databases (Swissprot, Nr, KEGG, InterPro, GO, and Pfam).

Type	Insert Size (bp)	Raw reads
Total	32,246	—
Nr (non-redundant)	23,872	74.03
InterPro (Protein sequence analysis & classification)	30,480	94.52
Swissprot (Swiss Institute of Bioinformatics and Protein Information Resource)	24,250	75.2
KEGG (Kyoto Encyclopedia of Genes and Genomes)	29,935	92.83
Pfam (The Pfam protein families database)	18,410	57.09
GO (Gene ontology)	23,644	73.32
Annotated	31,054	96.3

**Fig. 5:**
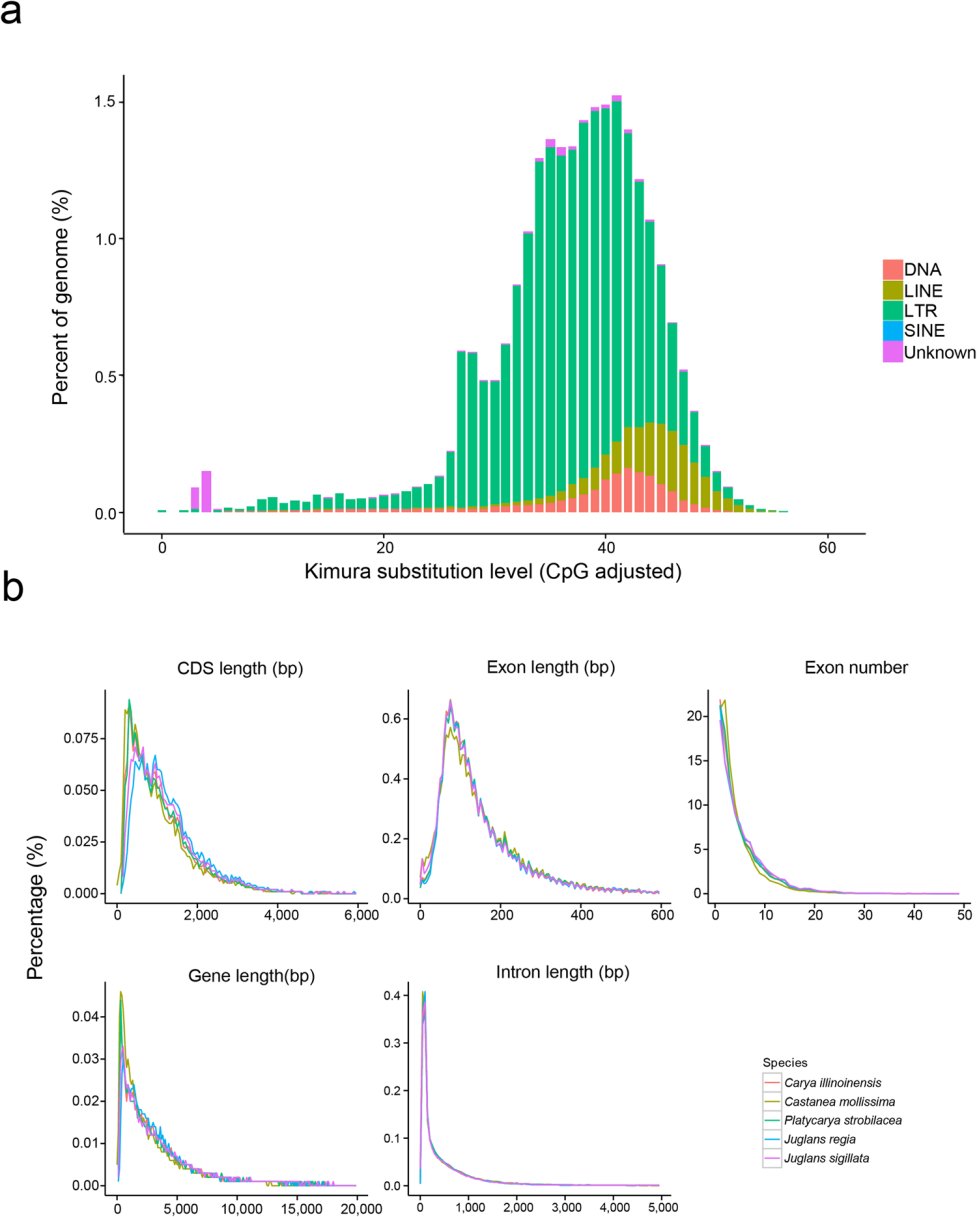
TE divergence distribution and genetic components of the *Platycarya strobilacea* genome and other four species. (**a**) TE sequence divergence distribution diagram. LINE = Long interspersed nuclear elements, LTR = Long terminal repeats, SINE = Short interspersed nuclear elements. (**b**) Comparison chart of CDS length, exon length, exon number, gene length, and intron length of *Platycarya strobilacea*, *Carya illinoinensis*, *Castanea mollissima*, *Juglans regia*, and *Juglans sigillata* genomes, respectively.

To identify transposable elements (TEs) and LTR-RTs (long terminal repeat retrotransposons) the *P. strobilacea* genome sequence was blasted against databases using Repbase v.20.05^38^, RepeatMasker v.4.0.7^39^, Tandem Repeats Finder (TRF) v4.09^40^, and PILER^41^, and LTRharvest v.1.5.10^42^ with the default parameters. The syntenic relationships within the species *P. strobilacea* were obtained using the MCSCANX software^43^. The final physical characteristics of the *P. strobilacea* genome assembly features were visualized using Circos^44^. We identified total of 271,999,812 bp (nearly half of the assembled genome length (41.72%)) of transposable element (TE) repetitive sequences in the genome assembly of *P. strobilacea* (NWU2021168) (Fig. 5a; Table 5). We detected the 31.24% of the genome length was occupied by e retroelement elements, constituting the predominant repeat type. The long terminal repeat (LTR) superfamily elements *Copia*, *Gypsy*, and DNA TEs constituted 223,145,245, 105,125,800, and 439,275,540 bp, corresponding to 32.95%, 15.52%, and 64.86% of the genome length, respectively. The density of *Copia* elements was twice as high as that of *Gypsy* elements in the *P. strobilacea* (NWU2021168) genome (Fig. 3b). We also annotated the non-coding RNA including transfer RNA (tRNA), ribosomal RNA (rRNA), small nuclear RNA (snRNA), and microRNA (miRNA) (Table 6). A total of 6,766 rRNA, 636 tRNA, 2,042 snRNA and 463 miRNAs were identified (Table 6). To validate genome annotation, we established the structure and number of genes in the *P. strobilacea* and four other species (*C. illinoinensis*, *C. mollissima*, *J. regia*, and *J. sigillata*) based on protein annotations from NCBI (Fig. 5b). A total of 32,246, 36,444, 31,074, 30624, and 30,387 protein-coding genes were identified in *P. strobilacea*, *C. mollissima*, *C. illinoinensis*, *J. regia*, and *J. sigillata*, respectively. The average length of the CDS, exon, gene, and intron in *P. strobilacea* was 1175.97 bp, 235.46 bp, 4,799.56 bp, and 902.18 bp, respectively (Fig. 5b). In addition, the average number of exons per gene was found to be equivalent across the five species.

**Table 5:** The statistical results of repeat sequences in *Platycarya strobilacea* genome.

Type	Denovo + Repbase		TE Proteins		Combined TEs	
	Length(bp)	% in Genome	Length(bp)	% in Genome	Length(bp)	% in Genome
DNA	7,733,421	1.12	327806	0.05	7,917,961	1.15
LINE	7,573,432	1.1	774281	0.11	8,063,715	1.17
SINE	9,790	0	0	0	9,790	0
LTR	240,768,286	34.99	95,928,887	13.94	257,450,664	37.41
Unknown	19,806,613	2.88	0	0	19,806,613	2.88
Total	271,999,812	39.53	97,030,905	14.1	287,061,345	41.72

**Table 6:** Abundance and size of noncoding RNA in *Platycarya strobilacea*.

Type		Copy number	Average length (bp)	Total length (bp)	Percentage of genome
miRNA		463	118.34	54,791	0.007962
tRNA		636	75	47,699	0.006932
rRNA	rRNA	3,383	286.85	970,427	0.140000
	18 S	359	1617.63	580,730	0.084394
	28 S	1,258	139.34	175,284	0.025473
	5.8 S	312	160	49,919	0.007254
	5 S	1,454	113.13	164,494	0.023905
snRNA	snRNA	1,021	110.89	113,220	0.016454
	CD-box	740	105.09	77,765	0.011301
	HACA-box	68	125.54	8,537	0.001241
	splicing	210	125.81	26,420	0.003839
	scaRNA	3	166	498	0.000072
	Unknown	0	0	0	0
		9,907	3143.62	2,269,784	32.88%

### Whole-genome duplication and subgenomes

**Fig. 6:**
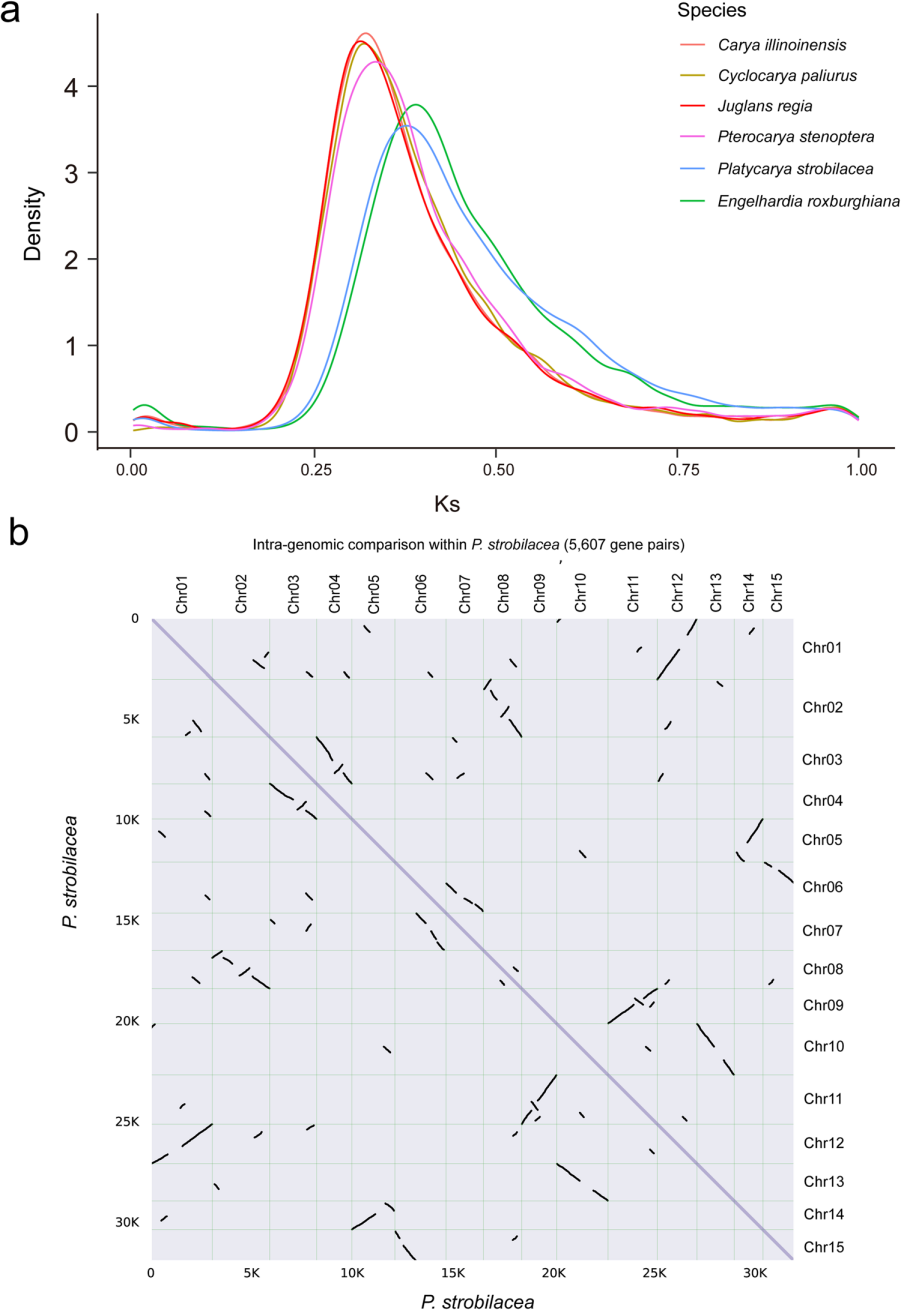
Whole-genome duplication (WGD) and subgenomes. (**a**) The Distribution of synonymous substitution rate (KS) for syntenic genes of *P. strobilacea* (light blue), *C. illinoinensis* (orange), *C. paliurus* (orange), *E. roxburghiana* (green), *J. regia* (red), and *P. stenoptera* (pink). The whole-genome duplication (WGD) events event was indicated by the peaks. (**b**) Dot-plot alignments within the assembled *P. strobilacea* chromosomes. The purple line and black dot-plot lines indicate homoeologous chromosomes within *P. strobilacea* genome. The dot-plot black line lines indicate paralogues produced by the whole-genome duplication event (WGD) and γ whole-genome triplication.

We calculated the whole-genome duplication (WGD) events using the software KaKs_Calculator v2.0^45^. The Ks distributions of orthologues among *P. strobilacea*, *C. illinoinensis*, *C. paliurus*, *E. roxburghiana*, *J. regia*, and *P. stenoptera* genomes were determined using the ggplot2 package^46^. We identified synteny and collinear blocks of genes using MCScanX^43^. We investigated the evidence for and the consequences of WGD in the *P. strobilacea* (NWU2021168) genome by comparing four Juglandaceae genomes (*Cyclocarya paliurus*, *Engelhardia roxburghiana*, *J. regia, C. illinoinensis, C. paliuru*, and *J. regia*), and the genome of *Vitis vinifera* (Fig. 6a). Paralogous relationships among the fifteen *P. strobilacea* genome chromosomes revealed seven main duplications representing subgenome (Fig. 3b), jointly containing 5,607 paralogous gene pairs in all collinearity blocks of the *P. strobilacea* genome (Fig. 6b). Both dot-plot alignments and paralogous blocks analyses showed seven main duplication subgenomes (chromosome pairs as follows: Chr1 and Chr12, Chr2 and Chr8, Chr3 and Chr4, Chr5 and Chr14, Chr6 and Chr7/Chr15, Chr9 and Chr11, and Chr10 and Chr13) within the assembled *P. strobilacea* chromosomes (Fig. 3b). We observed similar whole-genome duplication events (WGD) in the chromosomes 6, 7, and 15 (Fig. 3b). The synonymous nucleotide substitution (Ks) peak was ~0.3 within the *P. strobilacea* assembly, demonstrating that *P. strobilacea* experienced one mainly WGD event (Fig. 6a). Compared to the grape (*V. vinifera*) genome, *P. stenoptera* had one Ks small peak at ~0.9, which implied divergence between genes duplicated by the whole-genome triplication (γWGT). Previous studies showed that the genera *Engelhardia* and *Platycarya* are relatively ancient groups in the walnut family (Juglandaceae), which might have experienced an ancient WGD^5,6,10^.

## Data Records

The raw data (Illumina reads, PacBio HiFi reads, and Hi-C sequencing reads) used for genome assembly were deposited in the SRA at National Center for Biotechnology Information (NCBI)^47–49^. The RNA-seq data of eight tissues and organs female flower, male flower, mix female and flower inflorescence, axillary bud, new branch, stem, stem bark, and leaf were deposited in the SRA at NCBI SRR26346274- SRR26346281^50–57^. The final genome assembly files are deposited in NCBI Genbank^58^, and the final genome assembly and annotation files are available in Figshare^59^.

## Technical Validation

We assessed the completeness of genome assembly using Benchmarking Universal Single-Copy Orthologs (BUSCO)^28^ v3.0.2 with the default parameters. Gene families were selected for all-versus-all program BLASTP^16–20^. Based on the Hi-C sequencing reads, the scaffolds were anchored to 15 pseudomolecules using LACHESIS^26^.

## Data Availability

The data analyses were performed according to the manuals and protocols by the developers of corresponding bioinformatics tools and all software, and codes used in this work are publicly available, with corresponding versions indicated in Methods.

## References

[1] Yang YY, Qu XJ, Zhang R, Stull GW, Yi TS (2021). Plastid phylogenomic analyses of Fagales reveal signatures of conflict and ancient chloroplast capture. Mol Phylogenet Evol..

[2] Chen SC (2012). Geographic variation of chloroplast DNA in *Platycarya strobilacea* (Juglandaceae). J Syst Evol..

[3] Zhou ZK, Momohara A (2005). Fossil history of some endemic seed plants of east Asia and its phytogeographical significance. Acta Botanica Yunnanica..

[4] Cao Y (2023). Genomic insights into adaptation to Karst limestone and incipient speciation in East Asian *Platycarya* spp. (Juglandaceae). Mol Biol Evol..

[5] Manos PS, Stone DE (2001). Evolution, phylogeny, and systematics of the Juglandaceae. Ann Mo Bot Gard..

[6] Zhang Q (2021). Fossil-Informed models reveal a boreotropical origin and divergent evolutionary trajectories in the walnut family (Juglandaceae). Syst Biol..

[7] Lu AM (1982). On the geographical distribution of the Juglandaceae. Acta Phytotaxonomica Sinica..

[8] Wing SL, Hickey LJ (1984). The *Platycarya* perplex and the evolution of the Juglandaceae. Am J Bot..

[9] Fukuhara T, Tokumaru S (2014). Inflorescence dimorphism, heterodichogamy and thrips pollination in *Platycarya strobilacea* (Juglandaceae). Ann Bot..

[10] Manos PS (2007). Phylogeny of extant and fossil Juglandaceae inferred from the integration of molecular and morphological data sets. Syst Biol..

[11] Xiang X (2014). Large-scale phylogenetic analyses reveal fagalean diversification promoted by the interplay of diaspores and environments in the Paleogene. Perspect Plant Ecol..

[12] Zhou H (2020). Whole genome-based insights into the phylogeny and evolution of the Juglandaceae. BMC Ecol Evol..

[13] Mu XY (2020). Phylogeny and divergence time estimation of the walnut family (Juglandaceae) based on nuclear RAD-Seq and chloroplast genome data. Mol Phylogenet Evol..

[14] Wan Q (2017). Genetic divergence within the monotypic tree genus *Platycarya* (Juglandaceae) and its implications for species’ past dynamics in subtropical China. Tree Genet Genomes..

[15] Zheng Z (2014). East Asian pollen database: modern pollen distribution and its quantitative relationship with vegetation and climate. J Biogeogr..

[16] Marrano A (2020). High-quality chromosome-scale assembly of the walnut (*Juglans regia* L.) reference genome. Gigascience.

[17] Li X (2022). The Manchurian walnut genome: Insights into juglone and lipid biosynthesis. Gigascience.

[18] Yan F (2021). Improved de novo chromosome-level genome assembly of the vulnerable walnut tree *Juglans mandshurica* reveals gene family evolution and possible genome basis of resistance to lesion nematode. Mol Ecol Resour..

[19] Zhou H (2023). Pan-genome and transcriptome analyses provide insights into genomic variation and differential gene expression profiles related to disease resistance and fatty acid biosynthesis in eastern black walnut (*Juglans nigra*). Hortic Res..

[20] Ning DL (2020). Chromosomal-level assembly of *Juglans sigillata* genome using Nanopore, BioNano, and Hi-C analysis. Gigascience.

[21] Lovell JT (2021). Four chromosome scale genomes and a pan-genome annotation to accelerate pecan tree breeding. Nat Commun..

[22] Chen Y (2018). SOAPnuke: a MapReduce acceleration-supported software for integrated quality control and preprocessing of high-throughput sequencing data. Gigascience.

[23] Koren S (2017). Canu: scalable and accurate long-read assembly via adaptive k-mer weighting and repeat separation. Genome Res..

[24] Chin CS (2016). Phased diploid genome assembly with single-molecule real-time sequencing. Nat Methods..

[25] Li H, Durbin R (2009). Fast and accurate short read alignment with Burrows-Wheeler transform. Bioinformatics..

[26] Burton JN (2013). Chromosome-scale scaffolding of de novo genome assemblies based on chromatin interactions. Nat Biotechnol..

[27] Servant N (2015). Hic-Pro: An optimized and flexible pipeline for Hi-C data processing. Genome Biol..

[28] Simão FA (2015). BUSCO: Assessing genome assembly and annotation completeness with single copy orthologs. Bioinformatics..

[29] Stanke M (2006). AUGUSTUS: Ab initio prediction of alternative transcripts. Nucleic Acids Res..

[30] Slater GSC, Birney E (2005). Automated generation of heuristics for biological sequence comparison. BMC Bioinformatics..

[31] Holt C, Yandell M (2011). MAKER2: An annotation pipeline and genome-database management tool for second-generation genome projects. BMC Bioinformatics..

[32] Bairoch A, Apweiler R (2000). The SWISS-PROT protein sequence database and its supplement TrEMBL. Nucleic Acids Res..

[33] Pruitt KD, Tatusova T, Maglott DR (2005). NCBI Reference Sequence (RefSeq): a curated non-redundant sequence database of genomes, transcripts, and proteins. Nucleic Acids Res..

[34] Kanehisa M, Goto S (2000). KEGG: kyoto encyclopedia of genes and genomes. Nucleic Acids Res..

[35] Zdobnov E, Apweiler R (2001). InterProScan–an integration platform for the signature-recognition methods in InterPro. Bioinformatics..

[36] Ashburner M (2001). Gene Ontology: tool for the unification of biology. Nat Genet..

[37] Finn RD (2014). The Pfam protein family’s database. Nucleic Acids Res..

[38] Bao W, Kojima KK, Kohany O (2015). Repbase Update, a database of repetitive elements in eukaryotic genomes. Mobile DNA..

[39] Tarailo-Graovac M, Chen N (2009). Using Repeat Masker to identify repetitive elements in genomic sequences. Curr Protoc Bioinformatics..

[40] Benson G (1999). Tandem repeats finder: A program to analyze DNA sequences. Nucleic Acids Res..

[41] Edgar RC, Myers EW (2005). PILER: Identification and classification of genomic repeats. Bioinformatics..

[42] Ellinghaus D, Kurtz S, Willhoeft U (2008). LTRharvest, an efficient and flexible software for de novo detection of LTR retrotransposons. BMC Bioinformatics..

[43] Wang Y (2012). MCScanX: A toolkit for detection and evolutionary analysis of gene synteny and collinearity. Nucleic Acids Res..

[44] Krzywinski M (2009). Circos: an information aesthetic for comparative genomics. Genome Res..

[45] Wang D (2010). KaKs_Calculator 2.0: A toolkit incorporating gamma-series methods and sliding window strategies. Genom Proteom Bioinf..

[46] Kaori I, Murphy D (2013). Application of ggplot2 to pharmacometric graphics. Cpt-Pharmacometric Syst..

[47] (2023). NCBI Bioproject.

[48] (2023). NCBI Sequence Read Archive.

[49] (2023). NCBI Sequence Read Archive.

[50] (2023). NCBI Sequence Read Archive.

[51] (2023). NCBI Sequence Read Archive.

[52] (2023). NCBI Sequence Read Archive.

[53] (2023). NCBI Sequence Read Archive.

[54] (2023). NCBI Sequence Read Archive.

[55] (2023). NCBI Sequence Read Archive.

[56] (2023). NCBI Sequence Read Archive.

[57] (2023). NCBI Sequence Read Archive.

[58] (2024). NCBI Genome.

[59] Zhao P (2024). Figshare.

